# Emotional Body-Word Conflict Evokes Enhanced N450 and Slow Potential

**DOI:** 10.1371/journal.pone.0095198

**Published:** 2014-05-12

**Authors:** Jianling Ma, Chang Liu, Xin Zhong, Lu Wang, Xu Chen

**Affiliations:** 1 Key Laboratory of Cognition and Personality(SWU), Ministry of Education, Chongqing, China; 2 School of Psychology, Southwest University(SWU), Chongqing, China; 3 Yangtze Normal University, Chongqing, China; University of Leicester, United Kingdom

## Abstract

Emotional conflict refers to the influence of task irrelevant affective stimuli on current task set. Previously used emotional face-word tasks have produced certain electrophysiological phenomena, such as an enhanced N450 and slow potential; however, it remains unknown whether these effects emerge in other tasks. The present study used an emotional body-word conflict task to investigate the neural dynamics of emotional conflict as reflected by response time, accuracy, and event-related potentials, which were recorded with the aim of replicating the previously observed N450 and slow potential effect. Results indicated increased response time and decreased accuracy in the incongruent condition relative to the congruent condition, indicating a robust interference effect. Furthermore, the incongruent condition evoked pronounced N450 amplitudes and a more positive slow potential, which might be associated with conflict-monitoring and conflict resolution. The present findings extend our understanding of emotional conflict to the body-word domain.

## Introduction

Cognitive control refers to the ability to direct attentional resources toward task-related information while inhibiting task-irrelevant distractors. Such control is vitally important in the maintenance of normal cognitive functions. In the cognitive domain, this ability has commonly been examined using the classic Stroop color-word interference paradigm [1]. In this paradigm, participants are required to name the ink color of the color-word. The typical finding is a longer latency and more errors in the incongruent (e.g., when the word “red” is printed in blue ink) relative to the congruent (e.g., when the word “red” is printed in red ink) condition. Such behavioral differences are presumably due to competition for attentional resources by the conflicting stimuli in the incongruent condition [2].

The excellent temporal resolution offered by electroencephalography (EEG) may elucidate the dynamics of such conflictive processing. Indeed, two Stroop-related conflictive event-related potentials (ERP) have been observed. The first is the N450 [3]–[7], which has a negative polarity in incongruent minus congruent difference potentials with a latency of approximately 400–500 ms and centro-parietal topography. This component might be reflected in conflict detection or conflict resolution, as suggested by some researchers [4], [8]. Results of ERP source localization analysis suggest that the anterior cingulate cortex (ACC) might be the neural generator of the N450 [3], [4], [6]–[8]. Furthermore, researchers also found a sustained, conflict-sensitive slow potential (SP) following the N450. This potential has a parietal positivity/lateral frontal negativity beginning approximately 500 ms after stimulus onset [3], [9], [10] and was more positive following correct incongruent trials than congruent trials, suggesting its role in conflict resolution.

Given the recent evidence that cognitive control dysfunction in affective regions may be the main factor in emotional disorders, cognitive control in affective areas has received much attention. The most widely used paradigm to explore this is the emotional face-word Stroop task [11]–[13]. In this task, emotional facial expressions are superimposed with congruent (e.g., fearful facial expression with the word “fear”) or incongruent (e.g., fearful facial expression with the word “happy”) emotional words, and participants must respond to one emotional category and ignore the other [11], [13]. In a recent ERP study using this task, a negative-going component N350-550 and a positive-going component P700-800 in the incongruent minus congruent condition [12] was observed. The author argued that the N350-550 might reflect conflict resolution and was similar to N450 component, while the P700-800 resembled the slow potential, which was associated with post-response monitoring. This finding suggests that similar processing dynamics are involved in the resolution of conflict between an emotional face and an emotional word. Thus far, the neural dynamics of emotional conflict have been mainly investigated through the emotional face-word task, and it is unclear whether the aforementioned neural N450 and SP effect could be replicated in other emotional conflict tasks.

In the aforementioned emotional face-word Stroop task, emotional words are superimposed on emotional facial expressions, which are vital nonverbal signals of affective state in daily life. However, facial expression is only one means of nonverbally communicating emotional intentions. Recent studies have shown that body expressions also effectively convey affective intent [14]–[16]. Brain imaging studies provide evidence that body expressions evoke enhanced activity in brain structures involved in facial emotion processing [17]–[19]. Moreover, body expression increases activity in action representation areas [20]. Taken together, these findings strongly suggest that body expression is an important signal of affective state.

It has been repeatedly demonstrated that human brains respond to nonverbal affective signals from the face and body in a similar manner [21]. More specifically, behavioral evidence of these similarities comes from findings that threat signals conveyed by the body are detected as quickly as emotional facial expressions [22], [23]. Electrophysiologically, body expressions elicit robust emotional effects [24], [25] at a relatively early stage of processing. More specifically, the N170 has been found to be enhanced in response to affective body expressions relative to neutral body expressions [26]. Similarly, an inverted emotional body elicited larger N170 amplitudes than did an upright body [24]. In addition, the processing of fearful body expressions was associated with a shorter early P1 latency [25]. Moreover, emotional body expressions can modulate the activity of body-selective areas, just as emotional facial expressions modulate the activity of face-selective areas [27]–[29]. Recent findings from a functional brain imaging study showed that threat signals from both face and body increased activity in the amygdala [30]. In sum, these studies clearly show that, to some extent, analogous mechanisms are involved in the processing of emotional signals from the face and body.

Recent evidence suggests that emotional body expressions influence the processing of other emotional signals. More specifically, Van den stock et al. [31] found that, when both body and facial expressions were available, participants could easily categorize facial stimuli if both face and body conveyed the same emotion. When positive- and negative-valence body expressions served as contextual emotion cues, face memory accuracy was biased by this background information [32], [33]. Moreover, when an emotional face was presented within the context of an incongruent body posture (e.g., when a sad face was displayed on a fearful body), both adults' and 8-year-old children's perceptions of the emotional face were disrupted if the two emotions were highly similar (sad/fear), but not if they were highly dissimilar (sad/happy) [34]. These results indicate that the interaction between face and body depends on specific circumstances. A recent study [35] found that when processing angry body expressions, congruent face-body compound stimuli produced a persistently enhanced positive potential compared to incongruent stimuli, reflecting sustained attention towards and elaboration of threatening body expressions.

In addition, the perception of emotional body expressions is affected by other background signals of emotion. More specifically, a recent study found that when emotional body expressions were briefly presented as part of a social scene, they were better recognized when the actions in the background scene expressed an emotion congruent with the body expression [36], [37]. Interestingly, emotion signals from other irrelevant modalities may also affect body perception. For example, when dynamic whole-body expressions with a blurred face were presented in combination with happy or sad classical music, recognition of body language was much lower when the sad music was playing [38]. Moreover, even irrelevant human voices may bias body language perception when presented as background indicators of emotionality [39]. The shared characteristic of these background emotion signals is that they are all concrete. Meanwhile, numerous findings suggest that some abstract signals, such as emotional words, are also difficult to ignore when present in the background [40]. For example, in an emotional face-word task where participants were required to judge the emotion of facial stimuli, irrelevant emotional words influenced judgment performance [11], [13], [41]. Similarly, task-irrelevant words have been shown to decrease gender discrimination performance in a task in which participants must discern the gender of an emotional face [42], further demonstrating that emotional words in the background are difficult to inhibit. The abovementioned findings clearly indicate that while body expression perception may be affected by concrete background emotional information, it may also be influenced by task-irrelevant abstract emotional words. However, the latter has not yet been empirically examined.

In sum, there were two aims of the current study. First, based on the similarities in the processing of emotional facial and body expressions, we employed an emotional body-word Stroop conflict task, similar to the emotional face-word task, to explore the neural dynamics of emotional conflict processing, with the objective of replicating the N450/SP effect. Second, by using this task, we tested whether the perception of body expression could be influenced by background emotional words. Results derived from this new task may further our understanding of how conflicting emotional stimuli are processed and the extent to which emotional body expression perception is affected by other emotional signals. We expected that congruent body-word stimuli would be judged more quickly than incongruent body-word stimuli. Furthermore, we also anticipated an enhanced negative N450 ERP component and a more positive slow potential in response to the incongruent condition relative to the congruent condition.

## Methods

### Participants

Twenty-two undergraduate students took part in the pilot test of stimulus rating (11 female, *M*_age_ = 21.4 years, age range: 19–25 years). Another 25 students from the same participant pool (13 female, *M*_age_ = 22.6 years, age range: 20–25 years) participated in the formal ERP study. All participants were right-handed, had normal or corrected-to-normal vision, and had no history of psychiatric illness or neurological problems. Handedness was assessed through the Chinese version of the Edinburgh Handedness Inventory [43], [44]. None of the pilot study subjects participated in the formal ERP study. Written informed consent was obtained prior to conducting pilot or formal experiments, and all participants were appropriately compensated at the end of the experiments. The local ethics committee approved this study.

### Materials and procedure

Before the formal ERP experiment, we conducted one pilot validation test that included all of the body stimuli selected from the Bodily Expression Action Stimulus Test (BEAST) [45]. The BEAST consists of 254 grayscale images that each depict a whole-body expression of one of four emotions (64 anger, 67 fear, 61 happiness, and 62 sadness). The expressions are portrayed by 46 actors. The faces were blurred to avoid facial influence. The validation data for the BEAST showed that all of the emotions were recognized well; sadness was the easiest for participants to discriminate, followed by fear, anger, and happiness, which was most difficult. The BEAST has been successfully used in both healthy and clinical populations for behavioral, electrophysiological, and imaging studies. However, the BEAST validation process did not encompass pleasantness and arousal evaluation. In the first stage of our pilot test, each of the 254 stimuli was randomly presented for 4000 ms, with a 4000 ms inter-stimulus interval using E-Prime software. Twenty-two participants categorized the emotion expressed in the whole-body image by marking one out of four forced-choice alternatives (anger, fear, happiness, and sadness) on an answer sheet. Stimuli were presented in 3 blocks (100 pictures in each of the first two blocks and 54 in the last block) with a 5-min break between blocks. On average, sad expressions were most easily recognized (96% accuracy), followed by angry expressions (85%) and fearful expressions (83%). Happy expressions were most difficult to categorize. Since sad and angry expressions were better recognized than the other two expressions, we only used the stimuli depicting these two emotions as targets in the formal conflict task.

**Figure 1:**
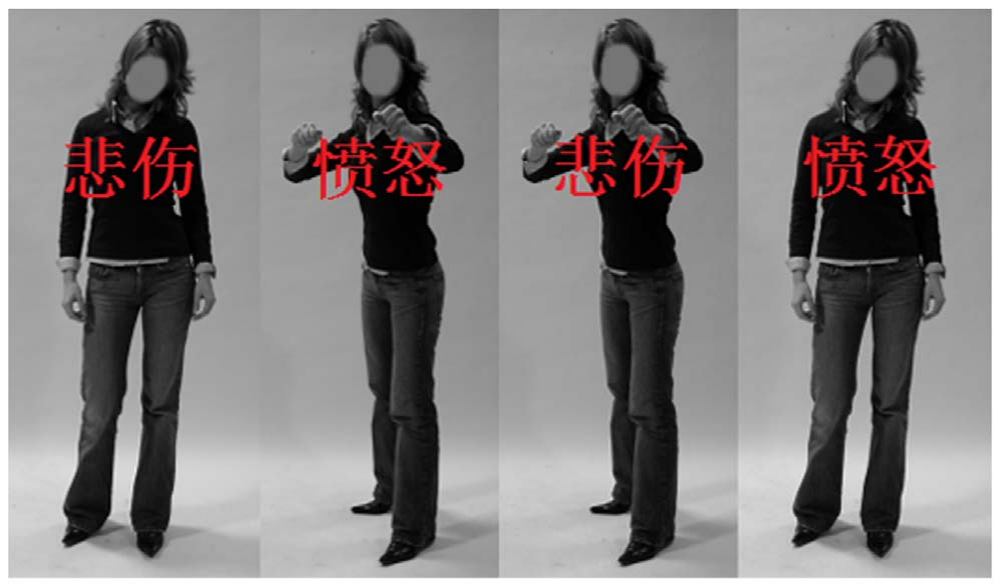
Examples of the four different categories body-word compound stimuli used in the experiment. Congruent and incongruent stimuli consisted of exactly the same material. The bodies of the two congruent stimulus conditions were swapped to create a mismatch and emotion expressed by the body and the word.

To avoid stimulus repetition, we randomly selected images of angry and sad expressions depicted by 20 different actors (10 female); the recognition accuracy associated with all selected images was above 80%. In summary, there were 20 angry and 20 sad body expression images selected. The mean recognition accuracy of these sad and angry body expressions was 0.94±0.07 and 0.92±0.05 respectively, and the accuracy did not differ significantly between the two categories, *t*(21) = 1.087, *p* = 0.469. Following ERP measurement, participants were required to rate the angry and sad body expressions in terms of arousal and pleasantness using a 9-point scale—pleasantness: 1 (*most pleasant*) to 9 (*most unpleasant*); arousal: 1 (*least arousing*) to 9 (*most arousing*). The results showed that angry body (5.29±0.68) was unpleasant than sad body (4.21±0.66), *t* (24) = 6.17, *p*<0.001; angry body (5.44±1.07) was much arousal than sad body (3.79±1.14), *t* (24) = 4.71, *p*<0.001. Examples of the stimuli used in the experiment are shown in Figure 1. These images were compounded with Chinese words using Photoshop. Two Chinese characters “愤怒” (“angry”) and “悲伤”(“sad”) were superimposed across the body in red color in 28-point Times New Roman font. A total of 80 compound images, 20 for each of the four types, were then made. The position of the word was fixed on the chest of the body in all of the body-word compound stimuli. Then, the four stimulus types (angry body-angry word, angry body-sad word, sad body-angry word, and sad body-sad word) were divided into congruent (body expression matched emotional word) or incongruent (body expression did not match emotional word) categories. The incongruent category was expected to induce conflict in emotion processing.

**Figure 2:**
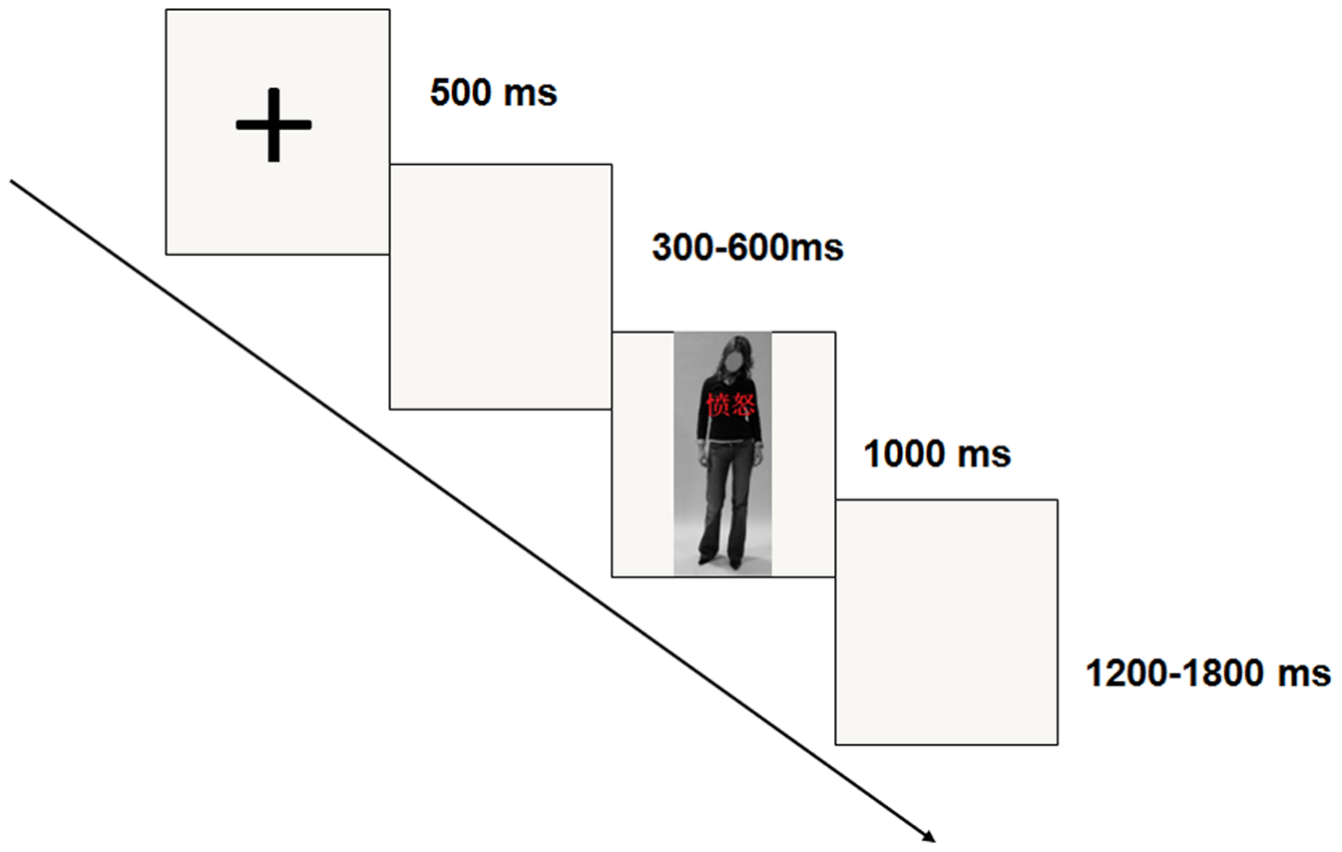
The order and timing of a trial is shown.

All of the compound stimuli were programmed and presented using E-Prime tools on a Dell 19-inch monitor. Participants were seated in a quiet room with dim light, and their eyes were at an 80-cm distance from the screen. The viewing angle of the framed body-word compound stimuli on the screen extended 9.87° vertically and 6.58° horizontally. Participants then completed an emotional-body-word Stroop task, in which they were required to identify the emotion of the body expression and ignore the meaning of the word superimposed across the body. Subjects were told to respond as quickly and accurately as possible by pressing “F” or “J” on the keyboard to indicate the expression of the image. Key allocations were counterbalanced across participants. All 80 compound stimuli were presented three times, generating 240 trials in total, which were equally separated into 3 blocks. Each block consisted of an equal number of congruent and incongruent trials. In each block, a trial began with a 500-ms fixation display, followed by a blank screen with an inter-stimulus interval that ranged from 300 to 600 ms. Then, the target body-word compound stimuli appeared on the center of the screen for 1000 ms; participants were required to respond within this time window. Following this, the buffer display was presented for 1200 to 1800 ms. Participants completed 20 practice trials before beginning the main task. During the individual experimental trial, participants were told to avoid blinking and other eye movements. The experimental procedure is illustrated in Figure 2.

### EEG recording

EEGs were recorded from each participant at 64 sites on the scalp using Ag/AgCl electrodes mounted in an elastic cap (Brain Products, GmbH, Germany) with references on FCz electrode site. Vertical electrooculograms (EOGs) were recorded using electrodes placed below the right eye. Horizontal EOGs were recorded from the right orbital rim. All inter-electrode impedances were maintained below 5 kΩ. Signals were amplified using a 0.01–100 Hz band pass filter and continuously sampled at 500 Hz/channel for off-line analysis.

### EEG analysis

Time domain analysis was performed by Brain Vision Analyzer (Brain Products, GmbH, Germany). All EEG signals were re-referenced off-line to TP9 and TP10 (average mastoid reference). The EEG data were digitally filtered with a 30 Hz low-pass filter and were epoched into a period of 1200 ms (200 ms baseline and 1000 ms post-stimulus onset). Signals were averaged across trials and time-locked to the onset of the compound stimuli separately for the congruent and incongruent categories. Trials with EOG artifacts (mean EOG voltage exceeding ±80 µv) and those contaminated with artifacts due to amplifier clipping, bursts of electromyography (EMG) activity, or peak-to-peak deflection exceeding ±80 µv were excluded from averaging. Averaged waveforms for each participant in each condition were calculated from the accepted trials.

**Figure 3:**
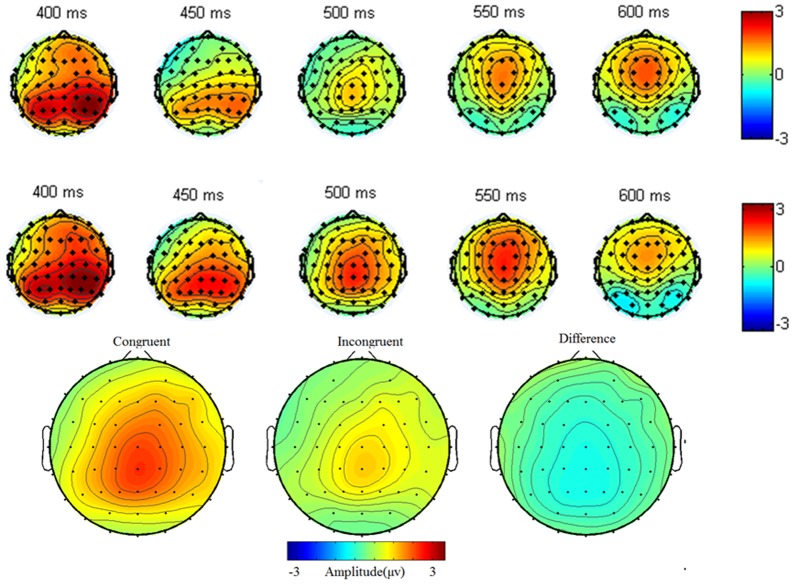
Top shows the topography distribution for incongruent (top upper) and congruent (top lower) compound stimuli between 400 and 600 ms from top view. Bottom shows scalp topography distribution of incongruent and congruent body-word at 500 ms and difference in their distribution.

The target ERPs were averaged separately from the point of their onsets. ERP and behavioral analyses were only conducted for trials in which the participant's response was correct; for these analyses, more than 100 trials were available for both congruent and incongruent conditions across subjects. Analysis of electrophysiological data focused on select electrode sites based on previous findings indicating that ERP modulations of interest are localized in frontal, central, central-parietal (N450; [3], [7], [12]), and posterior-parietal sites (SP; [3], [7], [12]), as well as the scalp topography distribution of the present study (Figure 3), which indicated maximal N450 and SP amplitudes over these areas. The N450 component was analyzed at nine sites: F1, Fz, F2; C1, Cz, C2; CP1, CPz, and CP2. These nine electrodes were divided into two factors: area (frontal, central, and central-parietal) and hemisphere (left, middle, right). Additionally, SP was analyzed at nine sites: CP1, CPz, CP2; P1, Pz, P2; PO3, POz, and PO4. These nine electrodes were divided into two factors: area (frontal, central, central-parietal, and parietal) and hemisphere (left, middle, right). The analysis time windows for N450 and SP were 400–500 ms and 600–800 ms, respectively. For both N450 and SP components, only the mean amplitude in the dedicated time window was analyzed. For both N450 and SP data, repeated-measures ANOVAs were conducted with congruency (congruent vs. incongruent condition), area (frontal, central, and central-parietal), and hemisphere (left, middle, and right) as within-factors. Statistics were adjusted using the Greenhouse-Geisser epsilon correction for nonsphericity if the number of factor levels exceeded two. Uncorrected degrees of freedom and corrected *p*-values are reported. If applicable, main effects were followed by pairwise comparison with Bonferroni correction. Reaction time and accuracy of the behavioral response were analyzed with a *t*-test.

## Results

### Behavioral results

Behavioral data showed a significant interference effect indicated by shorter latencies for congruent trials (*M* = 606 ms) compared to incongruent trials (*M* = 641 ms), *t* (24) = 5.061, *p*<0.001. Moreover, accuracy rates were higher for the congruent (*M* = 98%) compared to the incongruent (*M* = 96%) conditions, *t* (24) = 4.084, *p*<0.001.

### ERP results

#### N450 effect

**Figure 4:**
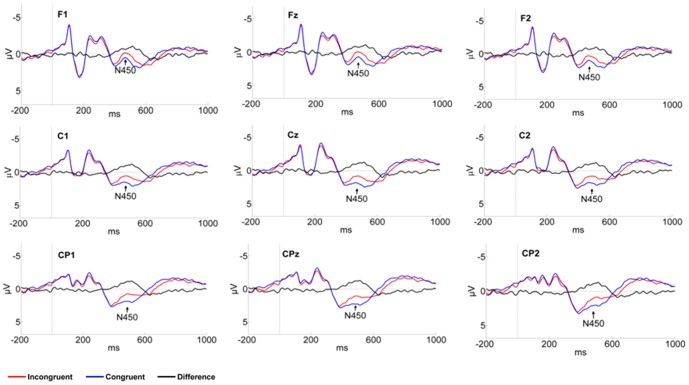
Group average ERP of N450 for congruent and incongruent compound stimuli at indicated electrode sites.

For mean N450 amplitude, results of the repeated-measures ANOVA showed a significant main effect of congruency, *F* (1, 24) = 14.687, *p*<0.001, η^2^_P_ = 0.38. As shown in Figure 4, the N450 corresponding to the incongruent body-word pair (*M* = 1.05 µv) was more negative than that associated with the congruent pair (*M* = 1.82 µv). There was also a main effect of area, *F* (2,48) = 13.645, *p*<0.001, η^2^_P_ = 0.362, and a Bonferroni correction comparison revealed that N450 amplitude at the central-parietal area (*M* = 1.92 µv) was more pronounced than at the central (*M* = 1.51 µv) and frontal areas (*M* = 0.88 µv). The main effect of hemisphere was also significant, *F* (2, 48) = 16.098, *p*<0.001, η^2^_P_ = 0.40. A Bonferroni correction comparison showed that N450 was enhanced in the right hemisphere (*M* = 1.61 µv) relative to the middle (*M* = 1.46 µv) and left (*M* = 1.24 µv) hemisphere. No other significant effects were found.

#### SP effect

**Figure 5:**
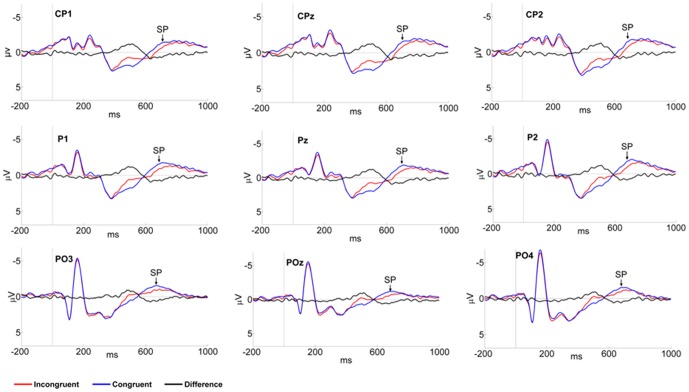
Group average ERP of SP for congruent and incongruent compound stimuli at indicated electrode sites.

For mean amplitude of the slow potential, the repeated-measures ANOVA showed a significant main effect of congruency, *F* (1, 24) = 14.88, *p*<0.001, η^2^_P_ = 0.383. As seen in Figure 5, the SP of the incongruent body-word pair (*M* = −0.71 µv) was more positive than that of the congruent pair (*M* = −1.18 µv). There was also a main effect of hemisphere, *F* (2,48) = 8.478, *p*<0.001, η^2^_P_ = 0.261, and a Bonferroni correction comparison revealed that SP amplitude was more pronounced in the right hemisphere (*M* = −1.07 µv) than in the middle (*M* = −0.86 µv) or left hemisphere (*M* = −0.92 µv). No other significant effects were found.

## Discussion

The aim of the present study was to investigate the neural dynamics of emotional conflict and explore how perception of emotional body expression is affected by other emotional signals. Our results are consistent with our hypothesis. Behavioral data revealed a robust interference effect, as indicated by the longer reaction time and greater error rate in the incongruent condition, indicating that the processing of emotional body expressions was affected by inconsistent emotional words. Moreover, electrophysiological data showed that the incongruent condition evoked an enhanced negative N450 amplitude and a more positive slow potential relative to the congruent condition. The significances of these findings is further discussed below.

Regarding the behavioral data, our findings are clearly in line with our hypothesis. That is, there were longer latencies and more errors associated with the incongruent relative to the congruent condition, indicating that irrelevant incongruent emotional words hamper, and irrelevant congruent emotional words facilitate, the categorization of emotional body expressions. Although previous studies [31], [32], [34], [46], [47] have shown similar behavioral effects using compound face-body and emotional face-word Stroop tasks in an emotional background scene, the present study demonstrates this effect in an emotional body-word task.

More importantly, the current behavioral conflict itself offers additional evidence for the importance of the body in the expression of emotion. Although the examination of facial-expression perception has dominated research in affective neuroscience, isolated facial expressions are never perceived in daily communication; rather, they are always accompanied by corresponding body cues [14], [16], [48]. In the past decade, increasing attention has been directed toward emotional body expression, and numerous studies provide compelling evidence for the role of the body in conveying emotional state [14]–[16], [20], [37], [45], [49]–[53]. For example, enhanced activity in the amygdala has been regarded as neural marker of emotional processing of facial expression; however, emotional body expressions have also been found to increase activity in this area [54]–[56]. In addition, participants perceive threatening body expressions more quickly than positive expressions within a neutral display [57], suggesting it may be adaptive to respond as fast as possible to dangerous signals in the environment. Considering that body expression can effectively convey emotional state, it has been referred to as emotional body language [14], [15].

Since the electrophysiological patterns associated with our emotional body-word task were highly similar to the classic Stroop-related potential, this new task may potentially represent a complementary tool for emotional conflict research. Analysis of the ERP data showed a significant N450 effect. This ERP effect was characterized by centro-parietal topography and negative polarity in incongruent minus congruent difference potential [58], [59]. Lines of study suggest that the N450 is sensitive to cognitive or emotional conflict monitoring and resolution between presented stimuli [7], [12], [60], [61]. Considering these explanations, the N450 within the current emotional body-word context may reflect conflict monitoring and conflict resolution between emotional body expression and the meaning of the presented emotional words. Moreover, as the N450 has been repeatedly replicated in the Stroop paradigm, not only in cognitive circumstance, such as classic Stroop experiment, numerical Stroop task [5], [9], [59], and cross-model Stroop-like [62] task, but also in emotional Stroop situation, it indeed can be acknowledged as a robust neural marker of conflict processing in the Stroop paradigm. Furthermore, the replicable occurrence of the N450 in Stroop studies may indicate that it is independent of some specific aspect of the current study set (e.g., both emotional body expressions and words bear negative valence). Previous ERP source localization attempts revealed that the ACC might be the neural generator of the N450 in cognitive and emotional Stroop tasks [3], [4], [7], [8], [12]. However, due to the limitation of volume conduction in EEG and the relatively low accuracy of the inverse method, this perspective should be accepted with great caution. Further examination is required to determine whether the body-word-conflict-associated N450 observed in the present study is generated in the ACC. Thus, future studies combining EEG/fMRI are needed to examine the association between the emotional conflict-related N450 and its underlying neural generator.

Following the N450 effect, there was a congruency effect between 600–800 ms. This effect had positive polarity in incongruent minus congruent potential difference and occurred over the parietal electrodes. Such a slow potential has been reported in previous cognitive and emotional Stroop studies [3], [4], [7], [12], [63] and is presumed to reflect conflict monitoring after response execution in emotional Stroop background tasks [12]. Lending support to this view, the SP in the current study may also signify conflict monitoring processing.

The current study shows similarities and differences when compared to previous studies using face-body compound stimuli to explore the dynamics of emotional integration from face and body expressions [35], [64]. Meeren et al. [64] found that P1 was the only neural marker of emotional congruency between facial expression and bodily expression at the early integration stage. The present results are similar to those of this study in that the stimuli in both studies are of negative valence; however, angry and fearful body and facial expressions were used in the previous study, while we adopted angry and sad body expressions and emotional words. Gu et al. [35] found that the integration of emotional signals from the face and body can be divided into three successive stages; while attentional focus has no effect on the first two stages, the third stage of integration is modulated by attentional fixation. The fundamental difference between our study and these two is that a Stroop-like paradigm was used in the present study. Rather, the prior studies focused on the integration of emotional signals from two sources: the effect of emotional facial expression on bodily perception, or bodily expression on facial perception. Consequently, they did not observe the typical Stroop-related conflictive potentials found in the present study.

In conclusion, the present study used an emotional body-word task and found a behavioral interference effect accompanied by a pronounced negative N450 and a more positive slow potential. These findings further our understanding of emotional conflict and suggest the emotional body-word task may effectively reveal the time course of emotional conflict.

## Supporting Information

Table S1 Behavioral data for 25 participants under congruent and incongruent conditions.
(DOC)

Table S2 N450 amplitudes data recorded from nine electrodes in the experiment.
(DOC)

Table S3 Slow potential amplitude data recorded from nine electrodes in the experiment.
(DOC)

Table S4 22 participants rate the selected out angry and sad body and the average accuracy.
(DOC)

Table S5 The average arousal and pleasant rating data of 25 participants for selected out angry and sad body expression.
(DOC)

File S1 Includes the entire body-word compound stimuli used in our experiment. All of emotional body stimuli were selected from De Gelder B, Van den Stock J (2011) The bodily expressive action stimulus test (BEAST). Construction and validation of a stimulus basis for measuring perception of whole body expression of emotions. Frontiers in psychology 2.
(RAR)
