# Supplementary material for: Emotional Body-Word Conflict Evokes Enhanced N450 and Slow Potential
Source: PLoS One. 2014 May 12;9(5):e95198. doi: 10.1371/journal.pone.0095198 (PMC4018289; doi:10.1371/journal.pone.0095198)
Supplement: Table S1 — Behavioral data for 25 participants under congruent and incongruent conditions. (DOC) [file pone.0095198.s001.doc]

Table S1. Behavioral data for 25 participants under congruent and incongruent conditions.

|  | **Latency** |  |  | **Accuracy** | |
| --- | --- | --- | --- | --- | --- |
|  | **Congruent** | **Incongruent** | | **Congruent** | **Incongruent** |
| Subject No. | average | average | Subject No. | average | average |
| 1 | 585.0666667 | 674.45 | 1 | 1 | 0.963333333 |
| 2 | 648.82 | 639.6833333 | 2 | 0.983333 | 0.97 |
| 3 | 595.3666667 | 661.2533333 | 3 | 1 | 0.94 |
| 4 | 770.65 | 836 | 4 | 1 | 1 |
| 5 | 538.94 | 542.5666667 | 5 | 0.973333 | 0.956666667 |
| 6 | 610.39 | 590.85 | 6 | 0.99 | 0.973333333 |
| 7 | 636.2633333 | 676.8666667 | 7 | 0.966667 | 0.973333333 |
| 8 | 614.2266667 | 720.84 | 8 | 0.956667 | 0.926666667 |
| 9 | 597.52 | 649.1333333 | 9 | 0.956667 | 0.95 |
| 10 | 573.45 | 607.76 | 10 | 0.966667 | 0.943333333 |
| 11 | 521.9433333 | 553.1833333 | 11 | 0.99 | 0.973333333 |
| 12 | 522.2533333 | 597.9633333 | 12 | 0.98 | 0.973333333 |
| 13 | 705.9666667 | 731.5533333 | 13 | 1 | 0.963333333 |
| 14 | 716.3133333 | 756.8766667 | 14 | 1 | 1 |
| 15 | 569.49 | 623.84 | 15 | 0.95 | 0.93 |
| 16 | 559.32 | 601.7966667 | 16 | 1 | 0.99 |
| 17 | 634.92 | 624.31 | 17 | 1 | 1 |
| 18 | 617.0133333 | 593.08 | 18 | 0.893333 | 0.913333333 |
| 19 | 670.58 | 726.3033333 | 19 | 0.973333 | 0.933333333 |
| 20 | 566.46 | 582.3266667 | 20 | 0.99 | 1 |
| 21 | 549.91 | 619.45 | 21 | 0.99 | 0.963333333 |
| 22 | 493.2433333 | 512.93 | 22 | 0.99 | 0.966666667 |
| 23 | 761.36 | 788.6266667 | 23 | 0.973333 | 0.973333333 |
| 24 | 532.0533333 | 522.8566667 | 24 | 0.99 | 0.983333333 |
| 25 | 569.6533333 | 595.1966667 | 25 | 1 | 0.99 |
