# Supplementary material for: Emotional Body-Word Conflict Evokes Enhanced N450 and Slow Potential
Source: PLoS One. 2014 May 12;9(5):e95198. doi: 10.1371/journal.pone.0095198 (PMC4018289; doi:10.1371/journal.pone.0095198)
Supplement: Table S2 — N450 amplitudes data recorded from nine electrodes in the experiment. (DOC) [file pone.0095198.s002.doc]

Table S2. N450 amplitudes data recorded from nine electrodes in the experiment.

|  | **F1** |  | **FZ** |  | **F2** |  | **C1** |  | **Cz** |  | **C2** |  | **CP1** |  | **CPz** |  | **CP2** |  |
| --- | --- | --- | --- | --- | --- | --- | --- | --- | --- | --- | --- | --- | --- | --- | --- | --- | --- | --- |
|  | **congruent** | **incongruent** | **congruent** | **incongruent** | **congruent** | **incongruent** | **congruent** | **incongruent** | **congruent** | **incongruent** | **congruent** | **incongruent** | **congruent** | **incongruent** | **congruent** | **incongruent** | **congruent** | **incongruent** |
| **Subjects No.** |  |  |  |  |  |  |  |  |  |  |  |  |  |  |  |  |  |  |
| 1 | -0.82039 | -1.3915 | -0.33598 | -0.95551 | 0.36231 | -0.42087 | 1.9644 | -0.32044 | 2.0039 | -0.27659 | 2.4391 | 0.32909 | 3.0349 | 0.42848 | 3.5646 | 0.7148 | 3.746 | 1.0609 |
| 2 | -0.72811 | -0.36254 | -0.69613 | -0.33259 | -0.26823 | -0.077937 | 0.08649 | -0.024956 | 0.30255 | 0.084386 | 1.1663 | 0.75047 | 0.6928 | 0.5104 | 1.006 | 0.66498 | 1.6166 | 1.1534 |
| 3 | 1.0539 | -0.21704 | 0.96532 | -0.021428 | 1.5937 | 0.83101 | 1.7389 | 0.64947 | 2.3529 | 1.1959 | 2.1204 | 1.2348 | 1.5444 | 0.21612 | 1.9789 | 0.65275 | 1.8102 | 0.57474 |
| 4 | 2.2286 | 1.7499 | 2.2635 | 1.7818 | 2.0449 | 1.6387 | 1.6849 | 1.194 | 1.7874 | 1.3458 | 1.6586 | 1.182 | 1.4273 | 1.044 | 1.3847 | 0.88284 | 1.415 | 0.80931 |
| 5 | 2.3587 | 2.3641 | 1.9419 | 2.0916 | 2.1822 | 2.3771 | 3.0095 | 2.8065 | 2.3763 | 2.2928 | 2.3042 | 2.2259 | 3.7236 | 3.6366 | 3.1561 | 3.2406 | 3.2375 | 3.2229 |
| 6 | 0.095814 | -0.30744 | 0.27589 | -0.24674 | 0.47904 | 0.087672 | 1.073 | 0.71676 | 1.1316 | 0.58732 | 1.7096 | 1.4101 | 2.1177 | 1.7601 | 2.5035 | 2.0398 | 2.7578 | 2.4342 |
| 7 | 2.0267 | 1.499 | 1.9967 | 1.2754 | 2.0766 | 1.7427 | 2.4141 | 2.0747 | 2.987 | 2.4103 | 2.688 | 2.1697 | 1.9262 | 1.7481 | 2.7272 | 2.2811 | 2.5435 | 2.1995 |
| 8 | -0.95683 | -0.91115 | -0.94495 | -0.88508 | -0.35261 | -0.72319 | -0.85766 | -0.70514 | -0.74662 | -0.65174 | -0.42018 | -0.49337 | 0.57444 | 0.026817 | 0.30769 | 0.044473 | 0.51584 | 0.13656 |
| 9 | -1.8612 | -2.6343 | -1.885 | -2.5458 | -1.6342 | -2.4158 | 8.81E-05 | -0.90354 | 0.081911 | -0.72514 | -0.10903 | -0.6916 | 1.1658 | 0.24944 | 1.3864 | 0.53443 | 1.0347 | 0.57093 |
| 10 | 1.6526 | 0.39977 | 1.918 | 0.54352 | 1.3144 | 0.24155 | 1.8762 | 0.49281 | 2.2498 | 0.72033 | 1.9022 | 0.46295 | 2.3064 | 0.8109 | 2.7274 | 1.0532 | 2.3317 | 0.89937 |
| 11 | 8.7321 | 8.3818 | 9.7051 | 9.2223 | 10.5818 | 10.0494 | 9.3076 | 8.4045 | 10.7391 | 9.6952 | 10.5561 | 9.7155 | 8.6614 | 7.8566 | 9.9138 | 8.9711 | 9.419 | 8.6628 |
| 12 | -1.5301 | -3.8278 | -1.6456 | -4.3201 | -0.92818 | -3.4833 | 1.844 | -0.2223 | 2.1703 | 0.088858 | 1.8289 | -0.226 | 2.4259 | 0.54342 | 2.852 | 0.9329 | 2.472 | 0.60655 |
| 13 | 0.62471 | 2.6776 | 1.0998 | 3.1258 | 1.553 | 3.1847 | 0.54533 | 2.7045 | 1.2109 | 3.6385 | 1.7332 | 3.5904 | 0.97676 | 3.152 | 1.659 | 4.0514 | 2.1065 | 4.0146 |
| 14 | 3.0347 | 1.4594 | 3.4699 | 1.6637 | 3.3584 | 1.6752 | 2.4576 | 0.94833 | 2.8103 | 1.1028 | 2.759 | 1.1553 | 1.8164 | 0.44735 | 1.9461 | 0.44689 | 2.2037 | 0.76651 |
| 15 | 1.9399 | 0.46535 | 1.8626 | 0.24762 | 2.1331 | 0.54136 | 2.9017 | 1.9288 | 3.4808 | 2.3832 | 3.1837 | 2.0567 | 3.1128 | 2.298 | 3.8725 | 2.9337 | 3.3922 | 2.471 |
| 16 | 3.8928 | 3.9391 | 3.9924 | 3.861 | 3.2854 | 3.0496 | 2.8613 | 3.1879 | 2.9648 | 3.1667 | 2.7889 | 2.7994 | 2.3263 | 2.7381 | 2.5368 | 2.8495 | 2.5164 | 2.6092 |
| 17 | 0.7201 | -0.7149 | 1.2827 | -0.69832 | 1.4018 | -0.027624 | 1.7471 | -0.51989 | 1.9203 | -0.6564 | 1.8216 | 0.073284 | 2.1973 | 0.20365 | 2.2935 | 0.03792 | 2.3588 | 0.3641 |
| 18 | 0.59004 | 0.0071199 | 0.64519 | -0.047804 | 0.76717 | 0.13124 | 0.83413 | 0.80029 | 1.0065 | 0.84442 | 0.89507 | 0.67229 | 0.92115 | 1.1819 | 1.139 | 1.1336 | 1.1145 | 1.1775 |
| 19 | 0.41417 | -0.61964 | 0.61554 | -0.75426 | 0.94078 | -0.4272 | 0.20988 | -1.439 | 0.3054 | -1.4178 | 1.0061 | -0.66918 | 0.85375 | -0.82662 | 1.424 | -0.44674 | 1.5679 | -0.061144 |
| 20 | 1.3985 | 1.4752 | 2.3149 | 2.0771 | 2.4531 | 2.9911 | 2.1757 | 2.1124 | 2.7734 | 2.8657 | 2.8461 | 2.8473 | 3.0308 | 2.8151 | 3.012 | 3.1397 | 3.2353 | 3.1251 |
| 21 | -0.24167 | -0.79683 | 0.24774 | -1.006 | 1.7062 | -0.23248 | 3.0934 | 1.6095 | 3.0921 | 0.23143 | 3.6655 | 0.57567 | 4.1656 | 2.9184 | 4.3207 | 1.7368 | 4.6138 | 1.8366 |
| 22 | 0.38173 | -1.3136 | 0.38651 | -1.2969 | 0.16726 | -1.0533 | 0.71997 | -1.0042 | 0.7018 | -0.81135 | 0.9387 | -0.36992 | 1.2919 | -0.24693 | 1.5553 | 0.081382 | 1.7209 | 0.52825 |
| 23 | -1.0937 | -1.201 | -1.1366 | -1.1751 | -1.0855 | -1.2135 | -1.6271 | -1.3295 | -1.6098 | -1.2575 | -1.2551 | -1.0953 | -1.0129 | -0.74364 | -0.93347 | -0.59594 | -0.7543 | -0.57302 |
| 24 | 0.64127 | 1.0517 | 0.93762 | 1.4828 | 1.2303 | 1.8424 | 1.3428 | 1.2681 | 2.0308 | 2.066 | 1.6573 | 2.2151 | 1.2668 | 1.08 | 1.5916 | 1.4042 | 1.1826 | 1.5748 |
| 25 | 0.20411 | -1.916 | 0.75883 | -1.502 | 1.264 | -0.92971 | 1.0612 | -1.204 | 1.9298 | -0.76841 | 1.9413 | -0.45727 | 1.98 | -0.24137 | 2.7742 | 0.17749 | 2.8394 | 0.42105 |
