# Supplementary material for: Emotional Body-Word Conflict Evokes Enhanced N450 and Slow Potential
Source: PLoS One. 2014 May 12;9(5):e95198. doi: 10.1371/journal.pone.0095198 (PMC4018289; doi:10.1371/journal.pone.0095198)
Supplement: Table S3 — Slow potential amplitude data recorded from nine electrodes in the experiment. (DOC) [file pone.0095198.s003.doc]

Table S3. Slow potential amplitude data recorded from nine electrodes in the experiment.

|  | **CP1** |  | **CPz** |  | **CP2** |  | **P1** |  |  | **Pz** |  |  | **P2** |  | **PO3** |  | **POz** |  | **PO4** |  |
| --- | --- | --- | --- | --- | --- | --- | --- | --- | --- | --- | --- | --- | --- | --- | --- | --- | --- | --- | --- | --- |
| **Subjects No.** | **Congruent** | **Incongruent** | **Congruent** | **Incongruent** | **Congruent** | **Incongruent** | **Congruent** | **Incongruent** | | **Congruent** | **Incongruent** |  | **Congruent** | **Incongruent** | **Congruent** | **Incongruent** | **Congruent** | **Incongruent** | **Congruent** | **Incongruent** |
| 1 | -1.8398 | -1.6388 | -2.2201 | -1.8901 | -2.2223 | -1.8793 | -2.9404 | -2.2006 |  | -2.9463 | -2.1331 |  | -3.0156 | -2.2817 | -1.9622 | -1.2874 | -1.9926 | -1.3698 | -1.8155 | -1.4938 |
| 2 | -0.27626 | 0.079543 | -0.58597 | -0.16504 | -1.0896 | -0.63725 | -0.93528 | -0.47688 |  | -1.1563 | -0.74077 |  | -1.7932 | -1.3398 | -1.4543 | -1.0842 | -1.4089 | -0.90649 | -1.8544 | -1.4491 |
| 3 | -1.1506 | -0.43595 | -1.2947 | -0.73308 | -1.6478 | -1.0185 | -1.5156 | -0.97386 |  | -1.332 | -0.8938 |  | -1.4765 | -0.97484 | -1.8078 | -1.5384 | -1.0654 | -0.97223 | -1.0725 | -1.2245 |
| 4 | -0.41118 | -0.25463 | -0.43643 | -0.14186 | -0.6769 | -0.13071 | -0.65273 | -0.34345 |  | -0.4541 | -0.22109 |  | -0.89728 | -0.33896 | -0.69642 | -0.54381 | -0.49009 | -0.26158 | -0.82485 | -0.47696 |
| 5 | -1.1798 | -1.8492 | -1.2112 | -1.8388 | -1.4916 | -1.9032 | -1.7472 | -2.3459 |  | -1.8347 | -2.2874 |  | -2.028 | -2.366 | -1.4007 | -1.7816 | -1.638 | -1.9307 | -1.9596 | -2.1639 |
| 6 | -1.0116 | -1.5448 | -1.14 | -1.6017 | -1.3753 | -1.8237 | -1.4738 | -1.9245 |  | -1.6862 | -1.9949 |  | -1.6831 | -2.0569 | -1.2267 | -1.4814 | -1.1327 | -1.4195 | -1.1443 | -1.4139 |
| 7 | -0.64194 | -0.28348 | -0.72606 | -0.31 | -0.54547 | -0.25975 | -0.79845 | -0.55918 |  | -0.3593 | -0.081517 |  | -0.19951 | -0.13411 | 0.23277 | 0.35717 | 0.93584 | 0.93577 | 0.50893 | 0.47064 |
| 8 | -0.20259 | 0.69126 | -0.1449 | 0.59143 | -0.32393 | 0.30455 | -0.78691 | 0.1444 |  | -0.85645 | 0.097414 |  | -0.88089 | -0.61241 | -1.2898 | -0.64661 | -0.61136 | -0.093543 | -1.2185 | -0.94468 |
| 9 | -0.53515 | -0.29664 | -0.81885 | -0.62655 | -0.73693 | -0.77889 | -1.0574 | -0.79543 |  | -0.97519 | -0.78761 |  | -1.1735 | -1.2623 | -0.92677 | -0.84871 | -0.72754 | -0.59688 | -1.1302 | -1.2076 |
| 10 | -1.9366 | -0.66443 | -2.2905 | -1.0052 | -2.0767 | -0.84258 | -2.4612 | -1.0156 |  | -2.2178 | -1.0539 |  | -2.4784 | -1.243 | -1.8427 | -0.75306 | -1.0599 | -0.39538 | -1.2976 | -0.50944 |
| 11 | -0.049762 | 0.043652 | 0.71843 | 0.2496 | 0.67904 | 0.22406 | -1.0254 | -0.81203 |  | -0.42661 | -0.61175 |  | -0.59449 | -0.65252 | -2.7043 | -2.1193 | -1.3633 | -0.96411 | -1.8554 | -1.2322 |
| 12 | -3.4523 | -1.0029 | -4.1937 | -1.7278 | -3.9132 | -1.7198 | -3.7164 | -1.3995 |  | -3.5282 | -1.4769 |  | -3.4695 | -1.6727 | -2.5027 | -1.0384 | -1.9667 | -0.5404 | -1.6932 | -0.76637 |
| 13 | -0.044673 | -0.62851 | -0.20911 | -0.676 | -0.74607 | -0.7197 | -0.25297 | -0.97284 |  | -0.11135 | -0.88037 |  | -0.80897 | -0.9793 | -0.31585 | -0.90677 | -0.11476 | -0.62109 | -0.87593 | -0.93862 |
| 14 | -1.504 | -0.67657 | -1.554 | -0.73971 | -1.6508 | -1.0333 | -0.70847 | 0.17476 |  | -0.86716 | -0.1378 |  | -0.866 | -0.30932 | 0.21952 | 0.82658 | 0.27864 | 0.69116 | 0.014654 | 0.21973 |
| 15 | -1.7888 | -1.6295 | -2.2477 | -1.9864 | -2.2462 | -1.9 | -1.7492 | -1.6294 |  | -2.0481 | -1.8644 |  | -2.165 | -1.8885 | -0.89894 | -0.80513 | -1.0592 | -0.89328 | -1.1205 | -0.82172 |
| 16 | -1.4421 | -1.2795 | -1.5642 | -1.3188 | -1.5378 | -1.2571 | -1.1986 | -1.1204 |  | -1.1954 | -1.0628 |  | -1.1981 | -0.9804 | -0.64918 | -0.74985 | -0.62012 | -0.64482 | -0.64464 | -0.4972 |
| 17 | -1.2964 | -0.20226 | -1.282 | -0.080051 | -1.5175 | -0.22594 | -1.7289 | -0.36798 |  | -1.2759 | -0.1267 |  | -1.7399 | -0.40265 | -1.1807 | -0.57978 | -0.76941 | 0.27108 | -1.3482 | -0.75343 |
| 18 | -1.2237 | -1.1799 | -1.411 | -1.1029 | -1.0577 | -1.0639 | -1.0807 | -1.313 |  | -1.076 | -1.0179 |  | -0.87297 | -1.0007 | -0.71785 | -0.7942 | -0.48704 | -0.53943 | -0.32357 | -0.51698 |
| 19 | -0.090445 | 0.46126 | -0.52994 | 0.21343 | -0.70861 | 0.10357 | -0.42986 | 0.2556 |  | -0.55015 | 0.0054505 |  | -0.93088 | -0.24838 | -0.076298 | 0.19197 | -0.21322 | 0.13671 | -0.64759 | -0.20787 |
| 20 | -3.7275 | -2.5948 | -3.6181 | -2.4553 | -3.8816 | -2.7907 | -3.9169 | -3.212 |  | -4.4332 | -3.3476 |  | -4.1598 | -3.2603 | -2.8078 | -2.3474 | -3.4417 | -3.1025 | -3.2154 | -2.922 |
| 21 | 2.358 | 4.2783 | 3.4336 | 4.7737 | 2.5438 | 3.4682 | 0.91406 | 3.1502 |  | 0.74853 | 2.8015 |  | 0.4555 | 2.0113 | -2.2752 | 0.029791 | -0.73012 | 0.94702 | -2.2437 | -0.94816 |
| 22 | -2.1216 | -1.0365 | -2.3304 | -1.3244 | -2.295 | -1.4745 | -1.5798 | -1.0674 |  | -2.0106 | -1.1743 |  | -2.2907 | -1.4687 | -0.70293 | -0.76686 | -0.65023 | -0.38337 | 0.089718 | -0.061973 |
| 23 | 0.55231 | 0.96488 | 0.51675 | 0.854 | 0.22878 | 0.63879 | -0.14168 | 0.16113 |  | -0.36426 | -0.12562 |  | -0.51649 | -0.3165 | -1.0273 | -0.79441 | -1.1277 | -1.1278 | -1.194 | -1.172 |
| 24 | -0.50255 | -0.61056 | -0.69938 | -0.69797 | -0.53173 | -0.79494 | -0.33926 | -0.27238 |  | -0.28793 | -0.167 |  | -0.058565 | -0.09705 | 0.10005 | 0.075352 | 0.044214 | 0.066708 | -0.11239 | -0.19417 |
| 25 | -0.41807 | 0.99608 | -0.86136 | 0.67942 | -1.2665 | 0.14805 | -1.391 | -0.080802 |  | -1.7727 | -0.28662 |  | -2.3701 | -0.9927 | -1.6926 | -0.69855 | -1.4878 | -0.5147 | -2.0866 | -1.131 |
