# Supplementary material for: Emotional Body-Word Conflict Evokes Enhanced N450 and Slow Potential
Source: PLoS One. 2014 May 12;9(5):e95198. doi: 10.1371/journal.pone.0095198 (PMC4018289; doi:10.1371/journal.pone.0095198)
Supplement: Table S4 — 22 participants rate the selected out angry and sad body and the average accuracy. (DOC) [file pone.0095198.s004.doc]

Table S4. 22 participants rate the selected out angry and sad body and the average accuracy.

|  |  | |  |  |  | |
| --- | --- | --- | --- | --- | --- | --- |
| **Subject No.** | **Angry body** | **sad body** | | |  |  |
| 1 | 0.97 | 0.863333333 | | |  |  |
| 2 | 0.92 | 1 | | |  |  |
| 3 | 0.916666667 | 1 | | |  |  |
| 4 | 0.966666667 | 0.936666667 | | |  |  |
| 5 | 0.943333333 | 1 | | |  |  |
| 6 | 0.916666667 | 0.8 | | |  |  |
| 7 | 0.916666667 | 1 | | |  |  |
| 8 | 0.9 | 1 | | |  |  |
| 8 | 0.81 | 0.8 | | |  |  |
| 9 | 0.953333333 | 0.963333333 | | |  |  |
| 10 | 0.88 | 1 | | |  |  |
| 11 | 0.829666667 | 1 | | |  |  |
| 12 | 1 | 1 | | |  |  |
| 13 | 0.953333333 | 0.863333333 | | |  |  |
| 14 | 0.87 | 0.866666667 | | |  |  |
| 15 | 0.953333333 | 1 | | |  |  |
| 16 | 0.826666667 | 0.863333333 | | |  |  |
| 17 | 0.916666667 | 1 | | |  |  |
| 18 | 0.993333333 | 0.896666667 | | |  |  |
| 19 | 1 | 0.95 | | |  |  |
| 20 | 0.9 | 0.903333333 | | |  |  |
| 21 | 0.953333333 | 1 | | |  |  |
| 22 | 0.98 | 0.92 | | |  |  |
