# Supplementary material for: Emotional Body-Word Conflict Evokes Enhanced N450 and Slow Potential
Source: PLoS One. 2014 May 12;9(5):e95198. doi: 10.1371/journal.pone.0095198 (PMC4018289; doi:10.1371/journal.pone.0095198)
Supplement: Table S5 — The average arousal and pleasant rating data of 25 participants for selected out angry and sad body expression. (DOC) [file pone.0095198.s005.doc]

Table S5. The average arousal and pleasant rating data of 25 participants for selected out angry and sad body expression.

| **arousal** | | |  | **pleasantness** | | |
| --- | --- | --- | --- | --- | --- | --- |
|  | **angry body** | **sad body** |  |  | **angry body** | **sad body** |
| **Subject No.** |  |  |  | **Subject No.** |  |  |
| 1 | 6 | 2.89 |  | 1 | 4.71 | 4.54 |
| 2 | 6.11 | 2.96 |  | 2 | 5.96 | 4.29 |
| 3 | 5.56 | 2.07 |  | 3 | 5.68 | 3.89 |
| 4 | 4.67 | 2.04 |  | 4 | 6.54 | 5.39 |
| 5 | 3.22 | 4.89 |  | 5 | 4.25 | 4.43 |
| 6 | 5.96 | 2.04 |  | 6 | 4.57 | 4.96 |
| 7 | 7.52 | 2.71 |  | 7 | 5.5 | 3.57 |
| 8 | 6.07 | 2.96 |  | 8 | 5.61 | 3.68 |
| 9 | 2 | 3.93 |  | 9 | 5.5 | 3.71 |
| 10 | 6 | 2 |  | 10 | 4.89 | 4.79 |
| 11 | 5.67 | 4.82 |  | 11 | 4.32 | 2.89 |
| 12 | 5.7 | 2.64 |  | 12 | 5.07 | 4.36 |
| 13 | 6.48 | 3.71 |  | 13 | 5.46 | 4.79 |
| 14 | 5.63 | 4.43 |  | 14 | 4.06 | 3.18 |
| 15 | 4.04 | 4.43 |  | 15 | 4.3 | 4 |
| 16 | 5.56 | 4.04 |  | 16 | 5.75 | 3.96 |
| 17 | 6.41 | 4.68 |  | 17 | 5.96 | 4.54 |
| 18 | 5.44 | 3.82 |  | 18 | 5.07 | 4 |
| 19 | 5.78 | 5.96 |  | 19 | 5 | 4.46 |
| 20 | 5.56 | 4.46 |  | 20 | 5 | 5 |
| 21 | 5.33 | 4.07 |  | 21 | 5 | 4.96 |
| 22 | 5.33 | 4.89 |  | 22 | 6 | 4.32 |
| 23 | 5.41 | 5.43 |  | 23 | 6 | 3 |
| 24 | 5.56 | 4.68 |  | 24 | 6 | 3.68 |
| 25 | 5.11 | 4.39 |  | 25 | 6 | 5 |
